# Strong selection pressures maintain divergence on genomic islands in Atlantic cod (*Gadus morhua* L.) populations

**DOI:** 10.1186/s12711-019-0503-5

**Published:** 2019-10-29

**Authors:** Silvia T. Rodríguez-Ramilo, Matthew Baranski, Hooman Moghadam, Harald Grove, Sigbjørn Lien, Mike E. Goddard, Theo H. E. Meuwissen, Anna K. Sonesson

**Affiliations:** 1 GenPhySE, INRA, 24 Chemin de Borde Rouge, 31326 Castanet-Tolosan, France; 2 Departamento de Mejora Genética Animal, INIA, Crta. A Coruña Km. 7,5, Madrid, 28040 Spain; 3 NOFIMA Marine, Osloveien 1, Ås, 1430 Norway; 4 Mowi ASA, Sandviksboder 77AB, Bergen, 5035 Norway; 5 Salmobreed, Sandviksboder 3A, Bergen, 5035 Norway; 6 Centre for Integrative Genetics, Department of Animal and Aquacultural Sciences, Faculty of Biosciences, Norwegian University of Life Sciences, Arboretveien 6, Ås, 1430 Norway; 7 Biosciences Research Division, Department of Economic Development, Jobs, Transport and Resources, Bundoora, VIC 3083 Australia; 8 Faculty of Veterinary and Agricultural Science, University of Melbourne, Parkville, VIC 3010 Australia; 9 Department of Animal and Aquacultural Sciences, Norwegian University of Life Sciences, Arboretveien 6, Ås, 1430 Norway

## Abstract

**Background:**

Two distinct populations have been extensively studied in Atlantic cod (*Gadus morhua* L.): the Northeast Arctic cod (NEAC) population and the coastal cod (CC) population. The objectives of the current study were to identify genomic islands of divergence and to propose an approach to quantify the strength of selection pressures using whole-genome single nucleotide polymorphism (SNP) data. After applying filtering criteria, information on 93 animals (9 CC individuals, 50 NEAC animals and 34 CC × NEAC crossbred individuals) and 3,123,434 autosomal SNPs were used.

**Results:**

Four genomic islands of divergence were identified on chromosomes 1, 2, 7 and 12, which were mapped accurately based on SNP data and which extended in size from 11 to 18 Mb. These regions differed considerably between the two populations although the differences in the rest of the genome were small due to considerable gene flow between the populations. The estimates of selection pressures showed that natural selection was substantially more important than genetic drift in shaping these genomic islands. Our data confirmed results from earlier publications that suggested that genomic islands are due to chromosomal rearrangements that are under strong selection and reduce recombination between rearranged and non-rearranged segments.

**Conclusions:**

Our findings further support the hypothesis that selection and reduced recombination in genomic islands may promote speciation between these two populations although their habitats overlap considerably and migrations occur between them.

## Background

Marine fish species are often distributed across a variety of habitats, which makes these organisms interesting models to study the interaction between gene flow and natural selection. Atlantic cod (*Gadus morhua* L.) exploits different ranges of salinity and temperature across an extensive geographical distribution. However, due to a large effective population size and to gene flow between habitats [1], a weak population genetic structure is predicted.

**Fig. 1:**
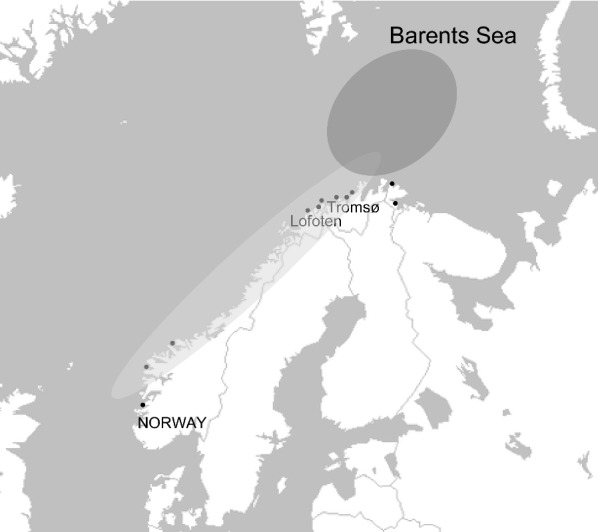
Geographical distribution of the wild CC (black points and light grey shading) and NEAC (dark grey shading) populations and the hybrid zone (where the distributions overlap) of individuals used for the National cod breeding program of Nofima in Tromsø (Norway)

Two extensively studied populations of Atlantic cod are the migratory Northeast Arctic cod (NEAC) population and the stationary population known as coastal cod (CC) [2, 3]. Generally, migratory individuals use deeper and more offshore habitats, and the NEAC animals migrate long distances from Lofoten, Norway, to the feeding areas in the Barents Sea. In contrast, stationary individuals usually occupy the Norwegian coastal water habitats during their entire life cycle. At present, NEAC is the largest population of Atlantic cod, and is located in the Barents Sea [3]. However, NEAC individuals can migrate to areas which are also inhabited by CC individuals (Fig. 1). Accordingly, breeding between NEAC and CC individuals occurs, but, to date, the degree of gene flow and interbreeding between both populations is not well known.

In addition to dissimilarities in migratory and feeding features, there are also clear differences in growth rate and age at maturity between these two populations [2]. Moreover, the CC population is less susceptible to viral nervous necrosis (VNN) than the NEAC population. Natural selection can explain these results, with the NEAC population being adapted to the Barents Sea environment, while the CC population is more resistant to pathogens that are present in a coastal environment [4]. Traditionally, differences in the structure of their otoliths have been used to differentiate individuals from either the NEAC or CC populations [5]. However, genetic differences between these two populations have also been identified (reviewed by [2]). On the one hand, differences have been found regarding blood type E [6], haemoglobin (*Hb*-*I*) alleles [7], 10 microsatellite loci [8], and alleles at the membrane protein gene *pantophysin* (*Pan I*) (now named *synaptophysin like 1* or *SYPL1*) [9]. At the *Pan I* locus, the frequency of the *Pan I*^*B*^ allele is above 90% in the NEAC population, whereas the *Pan I*^*A*^ allele predominates in the CC population [10]. On the other hand, little or no genetic differences between these populations have been detected at most allozymes [11], microsatellites [8] and at the mitochondrial cytochrome *b* locus [12]. It has been suggested that, during the early stages of divergence, genetic differentiation may have been restricted to a few specific genomic locations, called genomic islands, while the majority of the genome remained homogenised because gene flow is still in progress [13]. This has been supported by theoretical and empirical investigations [14]. Involvement of other mechanisms has been suggested, such as chromosomal rearrangements including inversions, divergence hitchhiking, and processes that promote the genomic co-localisation of genes [15]. In cod, several authors [3, 15–26] using different types of genomic data showed that population differentiation occurred at four discrete islands of genomic divergence located on different chromosomes (Table 1).

**Table 1:** Genomic data used and chromosomes on which genomic divergence was detected in previous studies

Study	Genomic data used	Chromosomes on which genomic divergence was detected
[3]	10,913 SNPs	1
[15]	1536 SNPs	1, 2, 7, 12
[16]	1641 SNPs	2, 7, 12
[17]	NGS of pooled DNA	1, 2, 7
[18]	491,265 SNPs	2, 7, 12
[19]	8165 SNPs	1, 2, 7, 12
[20]	8581 SNPs	1
[21]	8168 SNPs	1, 2, 7, 12
[22]	9187 SNPs	2, 7, 12
[23]	17 RFLP	Not indicated
[24]	594 SNPs	Not indicated
[25]	1536 SNPs	2, 7, 12
[26]	8809 SNPs	2, 7, 12

Recent developments have enabled next-generation sequencing technology to compare individual complete genomes with high precision. The objectives of our study were to identify genomic islands of divergence and to propose an approach to measure the strength of the natural selection within each genomic island in the NEAC and CC populations and their crosses using whole-genome single nucleotide polymorphism (SNP) data.

## Methods

### Genomic data

**Fig. 2:**
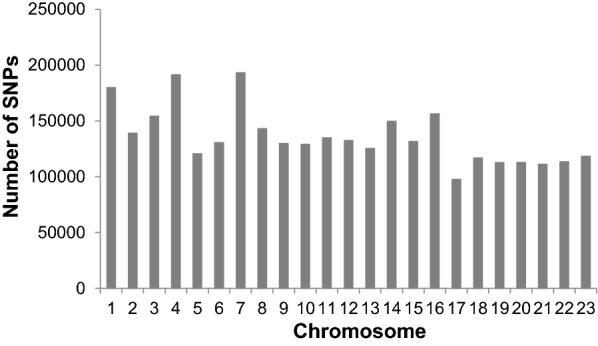
Number of SNPs analysed per chromosome

Genomic information from 111 animals belonging to the National cod breeding program of Nofima in Tromsø (Norway) was used. More specifically, year-classes (YC) from 2003, 2004 and 2005 were formed as progeny of wild Atlantic cod. Since the generation interval of Atlantic cod is 3 years, the data from YC 2006, 2007 and 2008 represent the first generation (F1) of the progeny of the selected fish from YC 2003, 2004 and 2005, respectively. Accordingly, the data from YC 2009 represent the second generation (F2) of the progeny of the selected fish from YC 2006. Each sire was mated to two dams, while each dam was mated to one sire. Furthermore, some sires and dams were also mated across YC to create genetic links between YC. The base population of the breeding program consisted of fish that were sampled from different geographical areas along the coast of Norway. The dataset consisted of fish with pure (CC and NEAC) and crossed origins (see [27, 28] for more details). Genomic DNA from these individuals was extracted for resequencing using the Truseq Library prep kit from Illumina (Illumina, San Diego, USA). Paired-end sequencing (2 × 100 nucleotides) was carried out using an Illumina HiSeq 2000 instrument to generate ~ 22× genome coverage for each sample. Reads were identified and filtered as follows: short reads were aligned against the cod genome assembly v 1.94 [29] using Bowtie 2 [30]. SAMtools [31] was then used to identify and retain uniquely mapped reads. SNPs were called using the FreeBayes (v0.9.18) software [32]. Finally, SNPs that were informative in more than 20% of the individuals and with a MAF higher than 0.05 were kept for further analysis. Individuals were classified based on their allelic differences at the *Pan I* locus [23] and their geographical origins. The final dataset included 93 animals (9 CC stationary individuals, 50 NEAC migratory animals, and 34 CC × NEAC crossed individuals) and 3,123,434 autosomal SNPs. Figure 2 shows the number of SNPs analysed for each chromosome.

### Genome-based estimates of coancestry

Following Malécot [33], it is possible to calculate the coancestry coefficient $f_{ij}$ between individuals $i$ and $j$. Genomic estimates of coancestry for each chromosome were obtained using the SNPs genotyped on each chromosome. Accordingly, genomic estimates of coancestry within chromosomes were obtained as follows. Because neighbouring SNPs showed significant coancestry differences, such that it was difficult to identify specific regions with a higher or lower coancestry based on individual SNPs, coancestry was estimated over sliding 200-SNP windows. Following this approach, the noisiness of single-locus coancestry estimates can be reduced and the precision of the estimates can be increased by combining data from several adjacent SNPs. This was based on the method proposed by Weir et al. [34]. For each chromosome, the first sliding window was identified by taking the first 200 SNPs at the beginning of the chromosome. Subsequently, the window slides across the chromosome by moving one SNP to the right, until the end of the chromosome is reached, maintaining 200 SNPs in each window. For each window, coancestry was estimated by taking the average of all coancestry values of the SNPs lying in that window.

Genome-based estimates of coancestry were obtained for the CC, NEAC, CC × NEAC populations, and we also calculated the coancestry coefficient $f_{{{\text{CC}}\;{\&}\;NEAC}}$ between the individuals of both populations.

### Coefficient of genetic differentiation

The Genepop software version 4.3 [35] was used to calculate Wright’s *F*_ST_ [36] between the CC and NEAC populations. *F*_ST_ was calculated by taking all the SNPs from each chromosome into account and also by using the same sliding window approach as above with a window size of 200 SNPs on each chromosome.

### Private allele frequency

Using the same sliding window approach, allele frequencies that were higher than 0 in the CC population and equal to 0 in the NEAC population, and vice versa, were averaged.

### Analysis of the statistical significance

**Fig. 3:**
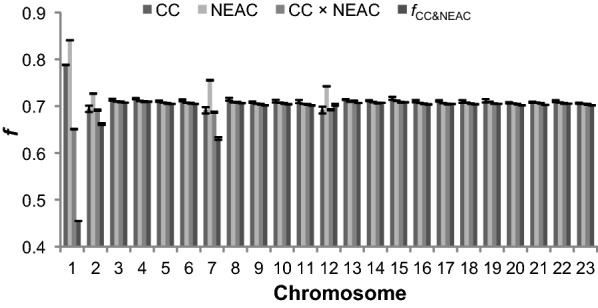
Mean coancestry coefficients ($f$) across chromosomes. Bars indicate standard errors

**Fig. 4:**
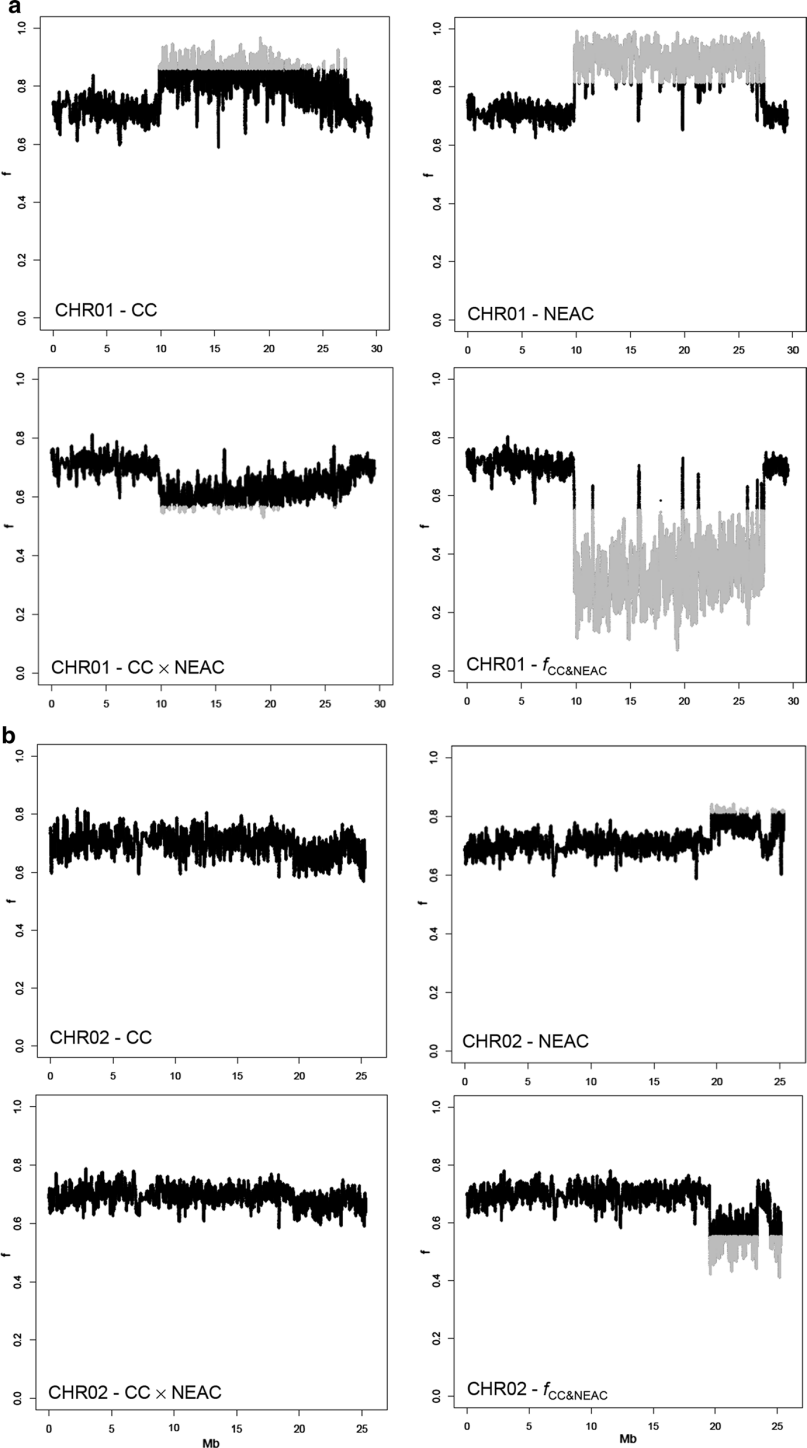

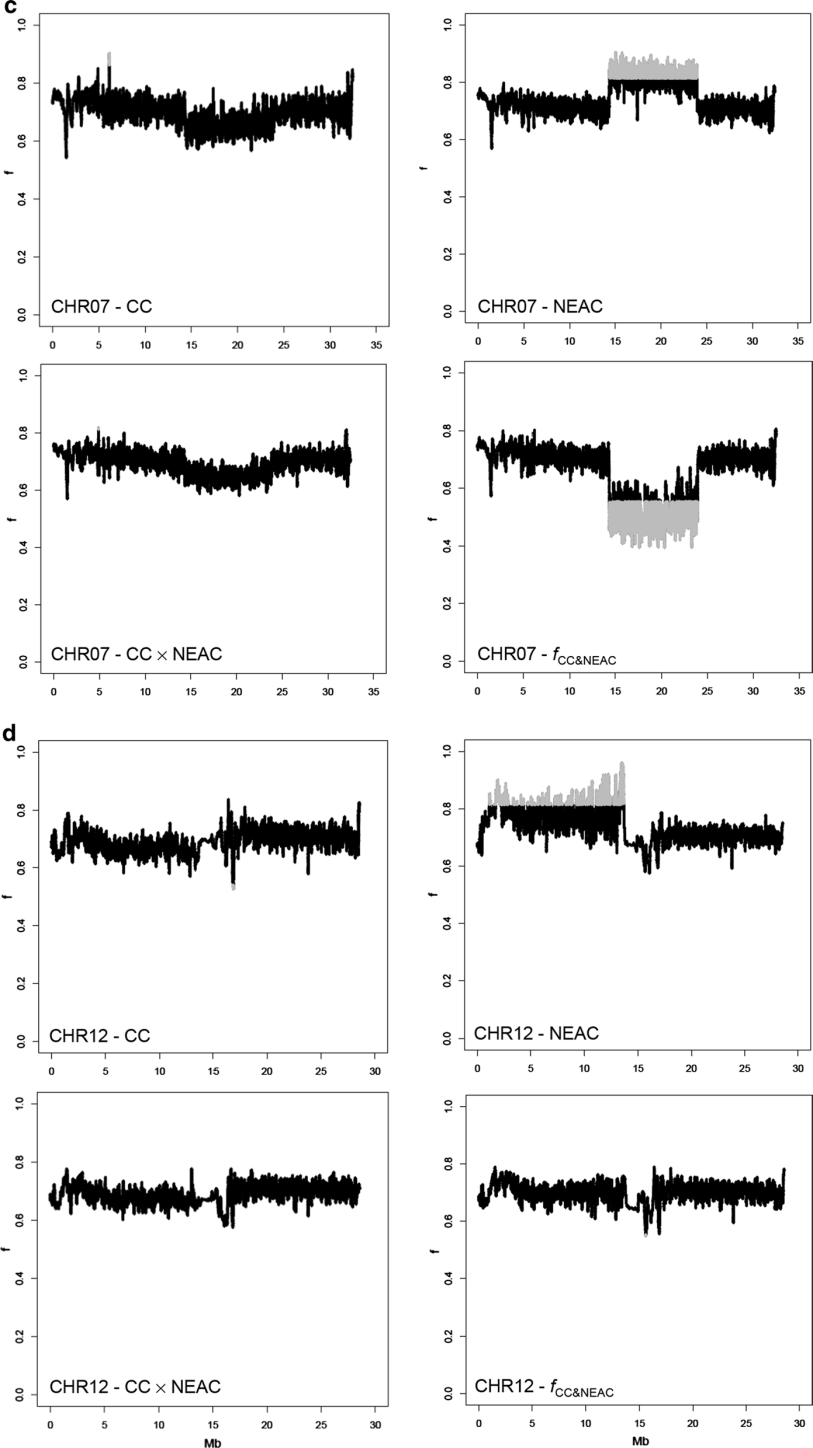
Patterns of coancestry ($f$) on chromosomes 1 (**a**), 2 (**b**), 7 (**c**) and 12 (**d**) for the CC, NEAC, CC × NEAC and $f_{{{\text{CC}}\;{\&}\;NEAC}}$ populations. Genomic islands with significantly higher or lower coancestry are shown in grey

**Fig. 5:**
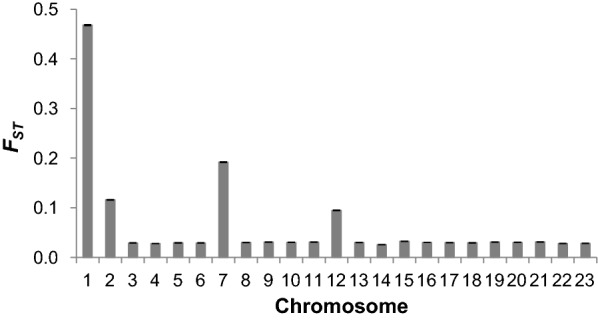
Mean genetic differentiation coefficients (*F*_ST_) across chromosomes. Bars indicate standard errors

**Fig. 6:**
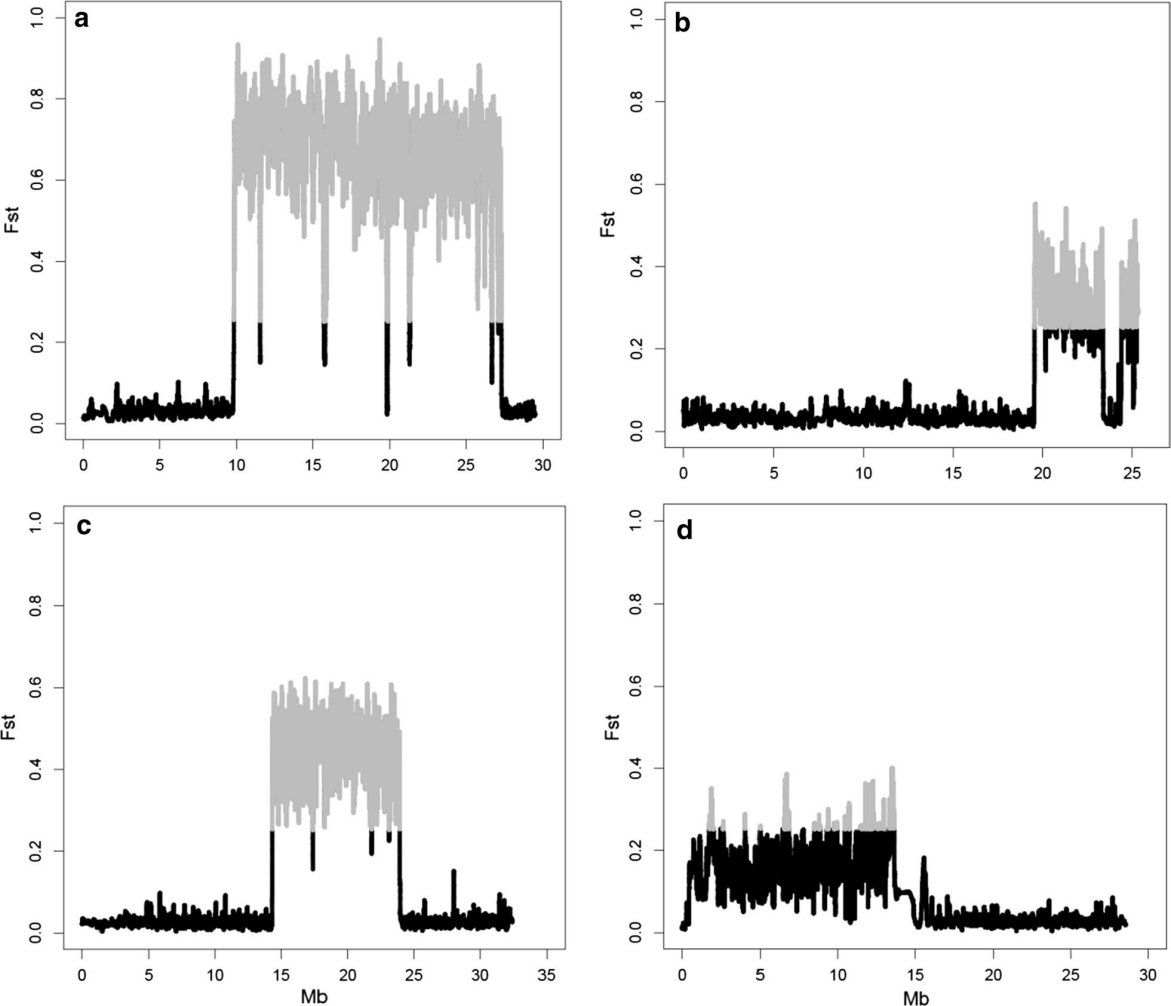
Genetic differentiation coefficient (*F*_ST_) between populations CC and NEAC for chromosomes 1 (**a**), 2 (**b**), 7 (**c**) and 12 (**d**). SNPs with a significantly higher differentiation coefficient are shown in grey

**Fig. 7:**
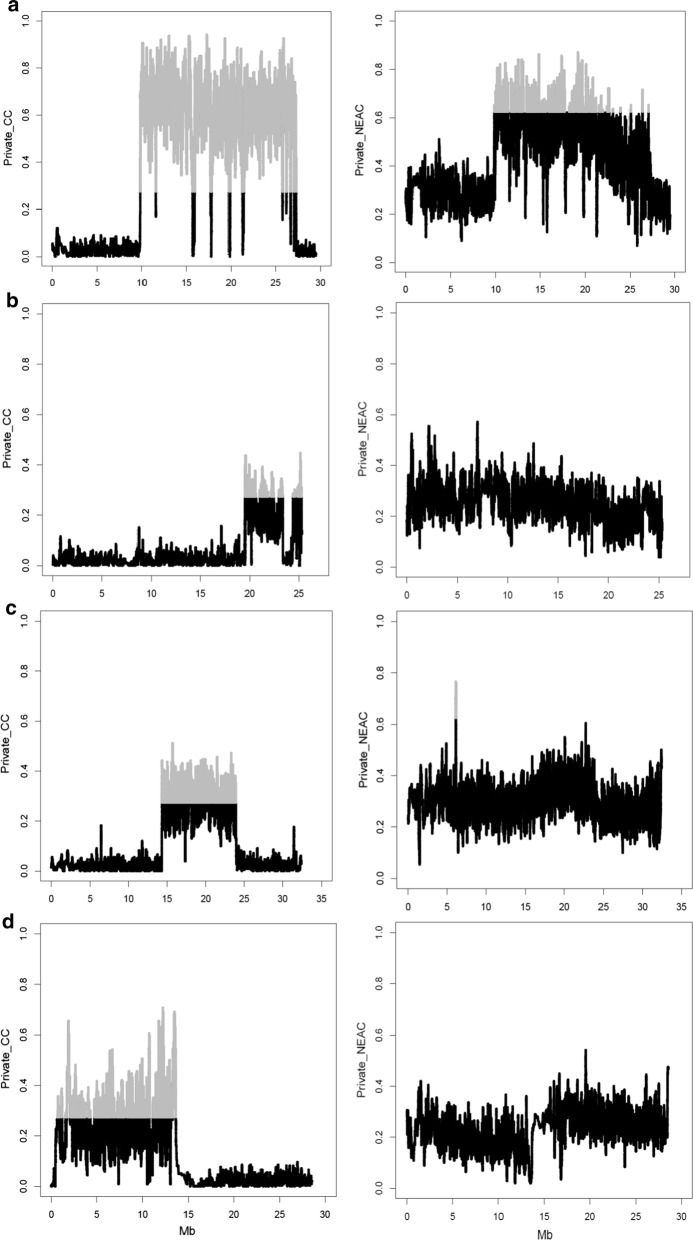
Private allele frequency in populations CC (left column) and NEAC (right column) on chromosomes 1 (**a**), 2 (**b**), 7 (**c**) and 12 (**d**). Markers with a significantly higher private allele frequency are shown in grey

The coancestry, the  genetic differentiation coefficients and the private allele frequency for all chromosomes (except for chromosomes 1, 2, 7 and 12, which clearly showed selection signatures; see Figs. 3 and 5) were used to establish the distribution of these values under the null-hypothesis. Significance thresholds were estimated as the 0.01% highest and lowest values for coancestry and as the 0.01% highest values for genetic differentiation and private allele frequency. Since the sliding windows overlap, the statistical tests were not completely independent, which reduced the effective number of tests performed, but this was not expected to affect the significance threshold. For instance, the threshold for the top 10 out of 1 million tests is approximately the same as for the top 1000 out of 100 million tests (for a large number of tests as was the case here). Although a P-value of 0.01% is very stringent, no explicit multiple testing correction was performed, such that these should be interpreted as nominal P-values. Nominally significant values are indicated in grey on Figs. 4, 6 and 7.

### Principal components analysis

The R-package Adegenet package version 2.0.1 [37] was used to calculate and plot the first two principal components based on the SNP data available for the genomic islands on chromosomes 1, 2, 7 and 12 and using all the SNP information for the other chromosomes.

An additional analysis was performed by classifying the individuals based on the PCA results. This analysis was performed for each genomic island on chromosomes 1, 2, 7 and 12. With this classification, it was possible to test the hypothesis that all the segregating SNPs in the group of homozygous animals for the rearranged segment were not segregating in the group of homozygous animals for the non-rearranged segment, and vice versa. In theory, SNPs that segregate in the group of animals with the rearranged segment should not segregate in the group with the non-rearranged segment because the rearrangement occurred on a single chromosome that later accumulated new mutations. However, these new SNPs do not segregate in the group of animals with the non-rearranged segment because the rearrangement prevents recombination between rearranged and non-rearranged segments.

Another analysis based on PCA results was performed to evaluate the possibility that the sampled fish resulted from recent crossbreeding between the CC and NEAC populations. In this situation, a rearranged segment on one chromosome would not be independent of the rearranged segment on the other chromosome. This is important because otherwise the use of the *Pan I* locus [23] to classify NEAC and CC individuals (as mentioned above) might bias the results on all the chromosomes and not just on chromosome 1. To test for this, we calculated the correlation between the rearranged alleles on different chromosomes, which was expected to be positive if the fish were recent crossbreds and vice versa.

### Estimation of selection pressure

The inbreeding coefficient of the two populations relative to the population from which they diverged for each locus $s$, $F_{s}$, can be estimated as:

$$F_{s} = \frac{{\left( {q_{1} - q_{2} } \right)^{2} }}{{q_{1} + q_{2} - 2q_{1} q_{2} }},$$

where $q_{1}$ and $q_{2}$ are the allele frequencies in the CC and NEAC populations, respectively. The average inbreeding ($F$) for all the chromosomes (except chromosomes 1, 2, 7 and 12; see Figs. 3 and 5) is calculated and $Nm$ (i.e. the product of effective population size $N$ by migration rate $m$) is estimated as:

$$Nm = \frac{{(1/\overline{F)} - 1}}{4}.$$

Accordingly, the average inbreeding within the genomic regions on chromosomes 1, 2, 7 and 12 (see Figs. 4, 6 and 7), is calculated. Based on these averaged inbreeding values and the above $Nm$ value, it is possible to estimate $Ns$ (i.e. the product of effective population size $N$ by selection pressure $s$) as:

$$Ns = \frac{{1 - \bar{F} - 4Nm\bar{F}}}{{2\left( {\bar{F} - 1} \right)}}.$$

$Ns$ are calculated separately for each of the chromosomes, i.e. 1 ($Ns_{1}$), 2 ($Ns_{2}$), 7 ($Ns_{7}$) and 12 ($Ns_{12}$). Accordingly, $Ns$ was calculated for each individual SNP (as mentioned above) and also for the entire rearranged segment based on the separation of individuals in rearranged and non-rearranged genotypes obtained from the PCA analysis. If $Ns$ > 1, selection is strong, and if $Ns$ < 1, selection is weak relative to genetic drift/inbreeding [38]. More details about the estimation of the selection pressure are provided in "Appendix".

## Results

### Estimates of coancestry

Mean coancestry across the genome did not differ significantly between CC (0.712 ± 0.004) and NEAC individuals (0.718 ± 0.006) (*P* = 0.425). The mean coancestry for the CC × NEAC individuals was smaller (0.702 ± 0.003), and the corresponding value of $f_{{\text{CC}} \; \& \; NEAC}$ was 0.689 ± 0.011.

Figure 3 shows that the largest differences in coancestry for the groups of evaluated animals were on chromosomes 1, 2, 7 and 12.

When coancestry values were smoothed by using a sliding window approach, differences in coancestry within chromosomes became clearer at specific genomic islands on chromosomes 1, 2, 7 and 12 (Fig. 4). A block of high coancestry in both the CC and NEAC populations was observed between 9 and 27 Mb on chromosome 1. In the same genomic island, the values of $f_{{\text{CC}} \; \& \; NEAC}$ indicate a genomic region that has a much lower coancestry coefficient. The patterns of coancestry on chromosomes 2, 7 and 12 in the NEAC population show islands of high coancestry between 18 and 25 Mb, 13 and 24 Mb, and 0 and 13 Mb, respectively. In these genomic regions on chromosomes 2 and 7, the values of $f_{{\text{CC}} \; \& \; NEAC}$ indicate regions of decreased coancestry.

More specifically, the genomic islands were between 8,819,361 bp and 27,328,570 bp, 18,352,060 bp and 25,309,797 bp, 13,344,692 bp and 23,924,283 bp and between 446,989 bp and 12,636,074 bp on chromosomes 1, 2, 7 and 12, respectively.

No pronounced blocks of coancestry were observed on the remaining chromosomes (see Additional file 1: Figure S1).

### Estimation of the genetic differentiation *F*_ST_

The mean genetic differentiation (*F*_ST_) value across the genome between the CC and NEAC populations was 0.062 ± 0.020. Using the SNPs that were genotyped on each chromosome, more precise genetic differentiation coefficients were obtained across the chromosomes (Fig. 5). The highest values of genetic differentiation between the CC and NEAC populations were observed on chromosomes 1, 2, 7 and 12.

Genetic differentiation coefficients for chromosomes 1, 2, 7 and 12 are in Fig. 6. *F*_ST_ values within each chromosome corroborate the genomic islands that were detected with the coancestry estimates. The most clear-cut genomic island with high *F*_ST_ values was on chromosome 1 also between 9 and 27 Mb, which indicates that alleles at this genomic island differ significantly between the CC and NEAC populations. Similar results were observed for chromosomes 2, 7 and 12.

No marked differences in genetic differentiation were observed for the remaining chromosomes (see Additional file 1: Figure S2).

### Private allele frequency

The analysis of private allele variants showed that the most differentiated chromosomes were also 1, 2, 7 and 12 (Fig. 7). However, patterns of private allele frequency contrasted with the coancestry patterns. For example, on chromosome 1, the most evident block of high coancestry was found for the NEAC population, whereas the most clear-cut block of private allele frequency was found for the CC population. This means that NEAC is the population with the original inversion. All initial alleles not carried by the rearranged block were lost in NEAC and most private alleles are therefore in CC. Similarly, the frequencies of private alleles for the regions on chromosomes 2, 7, and 12 were significantly increased in the CC population compared to the NEAC population. The reason is that if the private allele frequency is higher than 0 in the CC population, the other allele is fixed in the NEAC population.

No marked differences in patterns of private allele frequency were detected on the remaining chromosomes (see Additional file 1: Figure S3).

### Principal components analysis

**Fig. 8:**
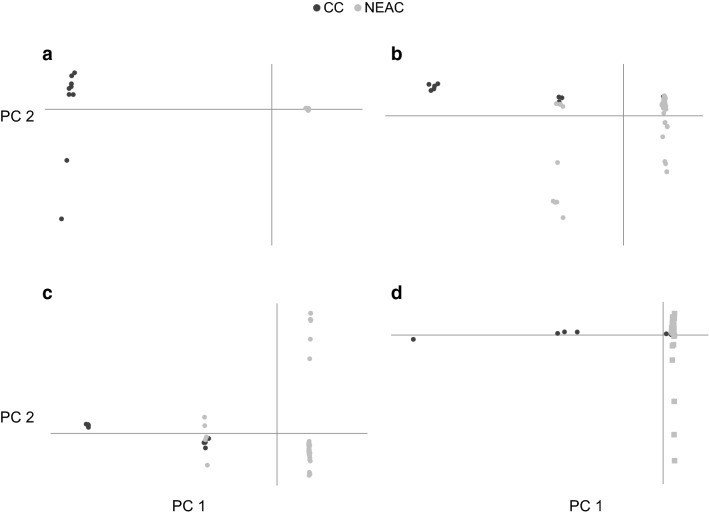
Principal components analysis within the genomic islands of chromosomes 1 (**a**), 2 (**b**), 7 (**c**) and 12 (**d**) for populations CC and NEAC

Principal components analysis showed differences between the two populations that formed completely separated clusters for chromosome 1, but these differences were less clear for chromosomes 2, 7 and 12 (Fig. 8).

No marked differences in the results of the principal components analysis were observed for the remaining chromosomes (see Additional file 1: Figure S4).

The PCA analysis clearly separated three groups of genotypes: (1) homozygous for the rearranged segment (i.e. the group of individuals that contains most of the NEAC individuals in the PCA analysis), (2) heterozygous for the rearranged segment (i.e. the group that includes both NEAC and CC individuals), and (3) homozygous for the non-rearranged segment (i.e. the group of individuals that contains most of the CC individuals in the PCA analysis) (Table 2).

**Table 2:** Classification of individuals after principal components analysis into homozygotes for the rearranged and non-rearranged segment and heterozygotes

Chromosome	1	2	7	12
Homozygotes for the rearranged segment	50 NEAC (100%)	41 NEAC (82%) and 1 CC (11%)	45 NEAC (90%)	50 NEAC (100%) and 5 CC (56%)
Heterozygotes		9 NEAC (18%) and 3 CC (33%)	5 NEAC (10%) and 5 CC (56%)	3 CC (33%)
Homozygotes for the non-rearranged segment	9 CC (100%)	5 CC (56%)	4 CC (44%)	1 CC (11%)

Individuals were classified based on their differences at the *Pan I* locus [23] and their geographical origins

Table 3 shows the total number of SNPs within each genomic island per chromosome and the segregating loci within each genomic island for the groups of animals with the rearranged and non-rearranged segment after the classification obtained from PCA. It is important to note that some SNPs segregate in both groups.

**Table 3:** Total number of SNPs in the genomic islands, number of SNPs segregating in the group of animals with the rearranged segment, the group of animals with the non-rearranged segment and in both groups for each chromosome

Chromosome	1	2	7	12
Total number of SNPs in the genomic island	132,995	52,160	90,446	65,915
Number of SNPs segregating in the rearranged group	23,126	14,824	17,103	37,129
Number of SNPs segregating in the non-rearranged group	39,338	7841	8923	0
Number of SNPs segregating both in the rearranged and non-rearranged groups	16,706	10,046	16,745	0

The coefficients of correlation between the rearranged and non-rearranged segments are in Table 4. Three of the four correlations were negative, which indicated that CC fish that carried one rearranged segment on one chromosome were unlikely to carry another rearranged segment on another chromosome. The values of the coefficient of correlation were not low, and the highest values were obtained for the negative correlation coefficients, which indicates that selection for a rearranged segment would not result in an increased fraction of rearranged segments on other chromosomes.

**Table 4:** Coefficients of correlation between the rearranged and non-rearranged segments

Pair of chromosomes	CC	NEAC
2 × 7	− 0.58	− 0.16
2 × 12	− 0.53	–
7 × 12	0.25	–

– Indicates that NEAC is fixed for the rearranged segment on chromosome 12

### Estimates of migration rates and selection pressures

**Fig. 9:**
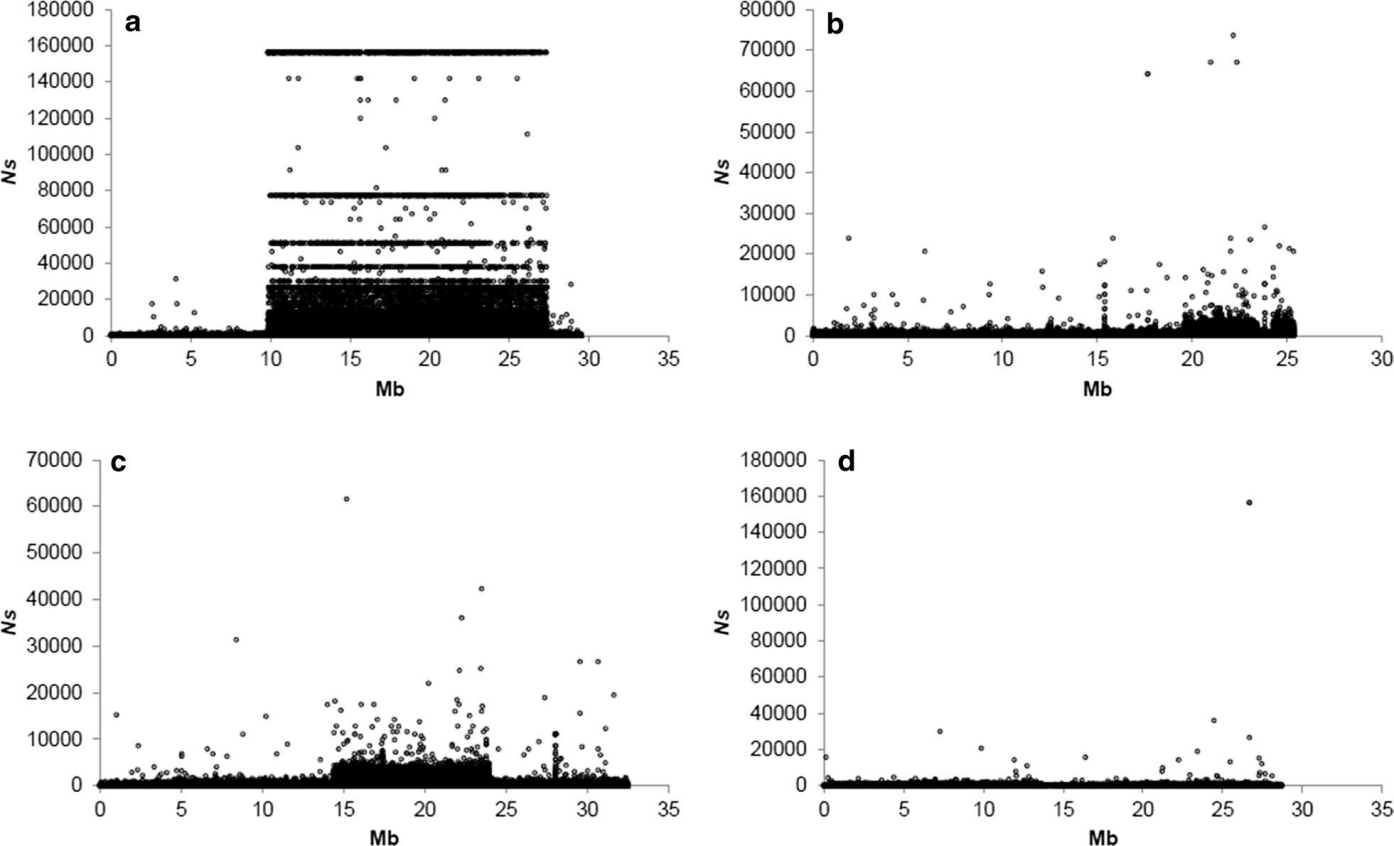
Estimates of the product of effective population size by selection pressure ($Ns$) for chromosomes 1 (**a**), 2 (**b**), 7 (**c**) and 12 (**d**)

The mean value of the product of effective population size $N$ by migration rate $m$, i.e. $Nm$ was equal to 4.37, which means that the contribution of migration is substantial compared to that of genetic drift. Based on the differences in allele frequencies for each SNP within each genomic island, the estimates of the product of effective population size $N$ by selection pressure $s$ were $Ns_{1} = 12.01$, $Ns_{2} = 2.26$, $Ns_{7} = 3.84$, and $Ns_{12} = 0.68$, for chromosomes 1, 2, 7 and 12, respectively, i.e., selection was strong relative to genetic drift in the genomic islands detected on chromosomes 1, 2 and 7, but not on chromosome 12 (Fig. 9). On chromosome 1, the same $Ns$ values were found for many SNPs along the genomic island (Fig. 9a). In addition, the highest values are quite far from the highest values obtained for the other three chromosomes. From the 3200 highest $Ns$ values found for this chromosome, 23 and 1616 correspond to private alleles in the CC and NEAC populations, respectively.

The resulting $Ns$ values for the entire rearranged regions were equal to 11.87, 15.42 and 2.86 for chromosomes 2, 7, and 12, respectively. Chromosome 1 was removed from this analysis because the genotypes at the *Pan I* locus (located on chromosome 1) were used to confirm the population identification, which would bias the allocation of non-rearranged regions to the CC and NEAC population. Thus, the selection pressures for the entire rearranged regions were higher than that for the individual SNPs within these regions.

## Discussion

In this study, genomic islands of divergence were identified and estimates of selection pressures in these genomic islands were obtained in two populations of Atlantic cod. The results indicate that natural selection is more important than genetic drift on these detected genomic islands of divergence.

The genomic islands of divergence identified on chromosomes 1, 2, 7 and 12 showed differences in $f$, which extended across 18, 7, 11 and 13 Mb, respectively for each chromosome, which suggest that these islands are considerably more related within populations and less related between populations than the rest of the genome (Fig. 4). Results based on the estimation of *F*_ST_ showed that the degree of divergence is higher for the chromosomes that carry these genomic islands (Fig. 5). However, it has been suggested that some factors can mask the detection of small genomic islands of divergence [39]. The mean whole-genome *F*_ST_ (0.062) estimated here agrees well with *F*_ST_ values from previous studies, which range from 0.024 to 0.065 [15, 24]. The increased frequencies of private alleles in these regions confirmed the general result that there is more population differentiation in these regions, which contain larger rearrangements that repress meiotic recombination in the NEAC × CC crosses [3, 21, 22].

The method used to estimate the migration rate between the two populations is well known and was previously described in the literature (e.g. [38]). A major assumption is that the allele frequencies have reached a balance between genetic drift (which is responsible for allele frequencies drifting apart) and migration (which reduces the differences in allele frequencies). In the case that these two forces are not (yet) in equilibrium and the populations are still drifting apart, migration will be overestimated, with the consequent implications in the estimation of the selection pressures. The estimation of the selection pressures in the genomic islands of divergence is a novel alternative approach, and is quite specific for the situation of a rearranged region. Assuming that there is no recombination between the non-rearranged and rearranged regions, selection will act on the rearrangement as a whole and thus cause the frequency of the rearrangement to differ between the populations.

The difference in frequency at individual SNPs is caused by the difference in the frequency of the rearrangement but the former is usually smaller than the latter. This is because the rearrangement will often carry the allele that is most common on the non-rearranged chromosome. Consequently, the frequencies of individual SNPs lead to underestimate the strength of selection. If some recombination occurs, the selection pressure on the causal loci will be stronger than that on the neutral loci, and the average selection pressure across the loci will further underestimate the selection pressure at the most important alleles that drive the divergence in these regions.

Assuming that a particular rearrangement only occurs once and does not recombine with the non-rearranged region, all the chromosomes that carry this rearrangement will descend from one original chromosome. Consequently, they will all carry the same alleles at each SNP within the rearrangement, and the original rearranged haplotype is thus monomorphic. Accordingly, the alternative region to the rearranged region will only occur in the wild-type haplotypes, and will be private to the wild-type haplotypes. This means that an increased private allele frequency points to wild-type haplotypes, which corresponds to the CC population, and the NEAC population corresponds to the rearranged haplotypes. It may be noted that the genome of NEAC individuals also has regions of increased private allele frequencies, which are due to mutations that occurred after the rearrangement. These later mutations may have drifted to considerable allele frequencies due to the originally low-frequency of the rearrangement, i.e. due to a hitchhiking effect.

The principal component analysis within the genomic islands revealed three genotypic groups (Fig. 8). For the genomic island on chromosome 1, these genotype groups match perfectly with the purebred NEAC and purebred CC population classification. For the other genomic islands, there is also a strong relationship between the population to which the individual belongs and the principal component classification. The classification of individuals into groups with and without the rearrangement from the principal components analyses highlighted that several SNPs segregate in both groups. There are three possible explanations for this. The first interpretation is that some individuals were erroneously allocated to the groups with and without the rearrangement. However, the principal components analysis showed a clear difference between the three groups. A second explanation is that the rearrangement was not a single event but happened several times. However, it seems unlikely that such a rearrangement occurred more than once at the same position in the genome. Finally, the most likely explanation is that a rearrangement will cause reduced recombination between the non-rearranged and rearranged alleles [22], but some recombinations will still occur, at a notably reduced rate, which is sufficient to introduce some segregating SNPs from the non-rearranged region into the rearranged region, and vice versa [40]. The analysis of the private allele frequency indicated that the ancestral alleles occur mainly in the CC population, and that the derived state occurs mainly in the NEAC population. After the rearrangement event, the recombination rate is reduced between the non-rearranged and rearranged alleles. Thus, the rearranged region could accumulate mutations that increase fitness in the habitat of the NEAC population, which is to the benefit of the migratory life-style. However, in other parts of the genome the allele frequencies of such mutants would be decreased due to the sustained introduction of migratory alleles at a rate of $Nm$ ~ 4 per generation.

Currently, $Ns$ values between 2.26 and 15.42 suggest that haplotypes are diverging, which results in two populations that coexist in an overlapping habitat but with different migratory behaviours. In fact, it has been suggested [16, 26] that the rearrangement on chromosome 1 is associated with migratory behaviour, whereas the rearrangements on chromosomes 2, 7 and 12 are most likely associated with temperature and/or salinity. Accordingly, the combination of these rearrangements could also have an effect on the migratory behaviour, temperature and/or salinity. The above situation may be an intermediate stage in the process of speciation of the two populations, where the fitness of the hybrid offspring is reduced, and consequently, the behavioural mating strategies that avoid matings with the wrong individuals will be favoured by natural selection. The latter results in a decreased migration rate and increases the divergence in the rest of the genome, which will promote the process of sympatric speciation throughout the genome. Hence the inverted chromosomal regions form the origin of a barrier to gene flow among populations that share a common habitat.

## Conclusions

Whole-genome SNP data for Atlantic cod were used to investigate genomic islands of divergence in the CC and NEAC populations. Our results show that gene flow between the populations was sufficient to limit divergence between the two populations except at four genomic islands. The high resolution of the SNP data used in this study enabled us to precisely locate four genomic islands of divergence on chromosomes 1, 2, 7 and 12 in the NEAC and CC cod populations and to estimate the natural selection pressures that lead to their divergence. The estimates of the selection pressures showed that natural selection was substantially more important than genetic drift in shaping the diverged regions on chromosomes 1, 2, 7, and 12.

## Supplementary information

**Additional file 1: Figure S1.** Patterns of coancestry ($f$) in all chromosomes (except 1, 2, 7 and 12) for the CC, NEAC, CC × NEAC and $f_{{{\text{CC}}\;{\&}\;NEAC}}$ populations. The data provided represent values of coancestry smoothed by using a sliding window approach. **Figure S2.** Genetic differentiation coefficients ($F$_ST_) between populations CC and NEAC in all chromosomes (except 1, 2, 7 and 12). The showed values are genetic differentiation coefficients smoothed by using a sliding window approach. **Figure S3.** Private allele frequency in populations CC (left column) and NEAC (right column) in all chromosomes (except 1, 2, 7 and 12). A sliding window approach was used to smooth the private allele frequency. **Figure S4.** Principal components analysis in populations CC and NEAC in all chromosomes (except 1, 2, 7 and 12). First two principal components on the SNP data available.

## Data Availability

The datasets analysed during the current study are available on reasonable request.

## Appendix

We will consider two populations CC and NEAC where some individuals migrate between the populations with a migration rate $m$. We will consider the homozygosity/inbreeding coefficient $F$ of the populations relative to when the populations diverged, at which time homozygosity was assumed 0. Consequently, crossbred CCxNEAC individuals are considered non-inbred, $F = 0$, and the same holds for crossbred chromosome segments that occur due to migration.

To this end we define the inbreeding coefficient $F$ relative to the crossbred (F1) population. The derivation of $F$ is very similar to that of $F$_ST_, except that the expected heterozygosity is taken in the aforementioned F1 population in order to express population differentiation (inbreeding) relative to this F1, i.e.

1 $$F = 1 - \frac{{O\left( {Het} \right)}}{{E\left( {Het} \right)}} = \frac{{(q_{1} - q_{2} )^{2} }}{{(q_{1} + q_{2} - 2q_{1} q_{2} )}}$$

where $O\left( {Het} \right)$ is the observed heterozygosity $\left[ {O\left( {Het} \right) = q_{1} \left( {1 - q_{1} } \right) + q_{2} \left( {1 - q_{2} } \right)} \right]$, and $E\left( {Het} \right)$ is the expected heterozygosity in the F1 $\left[ {E\left( {Het} \right) = q_{1} \left( {1 - q_{2} } \right) + q_{2} \left( {1 - q_{1} } \right) = q_{1} + q_{2} - 2q_{1} q_{2} } \right]$, where $q_{1} \left( {q_{2} } \right)$ is the allele frequency in population 1 (population 2). This $F$ statistic is very similar to $F$_ST_, and both statistics show a one-to-one relationship (result not shown).

Now we assume that individuals which are crossbred in a particular region have a reduced fitness by a factor of $\left( {1 - s} \right)$ where $s$ is the selection coefficient. Recombination within this region is assumed low, such that selection acts on the complete segment, and could not be directed against recombined parts of the segment (that e.g. carry particular loci that cause the fitness differences). The probability of a crossbred segment in generation $t$ is:

$$\left( {1 - F_{t} } \right) = \left[ {2m + \left( {1 - 2m - \frac{1}{2N}} \right)\left( {1 - F_{t - 1} } \right)} \right]\left( {1 - s} \right),$$

where $2m$ is the probability that one of the two gametes is a migrant; and $N$ is the effective population size; and $F_{t}$ is the $F$ statistic in generation $t$. We assumed here that the migrant segment hardly ever occurs in the homozygous state, and that the frequency of crossbred individuals is low, such that the overall population fitness remains close to 1. In addition, we assume that $s$ is small relative to 1, i.e. $\left( {1 - s} \right)$ is close 1, such that 1^st^ generation crossbreds do obtain (some) viable offspring, and a substantial part of the selection pressure against the migrant alleles occurs during later generations, when the migrant allele occurs in descendants from the original crossbreds. If the population is in equilibrium, i.e. $F_{t} = F_{t - 1} = F$, the above equation can be rewritten as (assuming $s$ is small relative to 1):

2 $$F = \frac{1 + 2Ns}{1 + 2Ns + 4Nm},$$

where $F$ is the inbreeding coefficient for the population in equilibrium.

In the absence of selection ($s = 0$), the above equation reduces to the well-known $F$ for populations in a migration-drift equilibrium [38]:

3 $$F = \frac{1}{1 + 4Nm}.$$

The latter is assumed to be the case for the majority of the genome outside the (rearranged) non-recombining regions.

Our strategy is thus to estimate the migration pressure, $4Nm$, by calculating average $F$ for all chromosomes, except the chromosomes carrying the rearranged, non-recombining regions using Eq. (1), and using Eq. (3) to obtain $4Nm$. Next, average $F$ is calculated within each of the non-recombining regions, and this $F$ and the aforementioned $4Nm$ estimate are used to obtain $Ns$ from Eq. (2). Estimates of $Ns > > 1$ indicate that the selection against the migrant chromosome segments is much stronger that the population genetic drift, and $Ns < < 1$ indicates that selection against these chromosome segments is an unimportant genetic force relative to the genetic drift.

## References

[1] Waples R (1998). Separating the wheat from the chaff: patterns of genetic differentiation in high gene flow species. J Hered..

[2] Nordeide JT, Johansen SD, Jørgensen TE, Karlsen BO, Moum T (2011). Population connectivity among migratory and stationary cod *Gadus morhua* in the Northeast Atlantic-a review of 80 years of study. Mar Ecol Prog Ser.

[3] Kirubakaran TG, Grove H, Kent MP, Sandve SR, Baranski M, Nome T (2016). Two adjacent inversions maintain genomic differentiation between migratory and stationary ecotypes of Atlantic cod. Mol Ecol.

[4] Ødegård J, Sommer A, Præbel AK (2010). Heritability of resistance to viral nervous necrosis in Atlantic cod (*Gadus morhua* L.). Aquaculture..

[5] Rollefsen G (1933). The otoliths of the cod—preliminary report. Fiskeridir skr..

[6] Møller D (1966). Genetic differences between cod groups in the Lofoten area. Nature.

[7] Dahle G, Jørstad KE (1993). Haemoglobin variation-a reliable marker for cod (*Gadus morhua* L.). Fish Res..

[8] Westgaard JI, Fevolden SE (2007). Atlantic cod (*Gadus morhua* L.) in inner and outer coastal zones of northern Norway display divergent genetic signature at non-neutral loci. Fish Res..

[9] Fevolden SE, Pogson GH (1997). Genetic divergence at the *synaptophysin* (*Syp I*) locus among Norwegian coastal and north-east Arctic populations of Atlantic cod. J Fish Biol.

[10] Sarvas TH, Fevolden SE (2005). Pantophysin (*Pan* I) locus divergence between inshore v. offshore and northern v. southern populations of Atlantic cod in the northeast Atlantic. J Fish Biol..

[11] Mork J, Ryman N, Stahl G, Utter F, Sundnes G (1985). Genetic variation in Atlantic cod (*Gadus morhua*) throughout its range. Can J Fish Aquat Sci.

[12] Árnason E (2004). Mitochondrial cytochrome b DNA variation in the high-fecundity Atlantic cod: Trans-Atlantic clines and shallow gene genealogy. Genetics.

[13] Nosil P, Funk DJ, Ortiz-Barrientosw D (2009). Divergent selection and heterogeneous genomic divergence. Mol Ecol.

[14] Via S (2012). Divergence hitchhiking and the spread of genomic isolation during ecological speciation-with-gene-flow. Philos Trans R Soc Lond B Biol Sci.

[15] Hemmer-Hansen J, Nielsen EE, Therkildsen NO, Taylor MI, Ogden R, Geffen AJ (2013). A genomic island linked to ecotype divergence in Atlantic cod. Mol Ecol.

[16] Bradbury IR, Hubert S, Higgins B, Borza T, Bowman S, Paterson IG (2010). Parallel adaptive evolution of Atlantic cod in the eastern and western Atlantic Ocean in response to ocean temperature. Proc Biol Sci..

[17] Karlsen BO, Klingan K, Emblem Å, Jørgensen TE, Jueterbock A, Furmanek T (2013). Genomic divergence between the migratory and stationary ecotypes of Atlantic cod. Mol Ecol.

[18] Barney BT, Munkholm C, Walt DR, Palumbi SR (2017). Highly localized divergence within supergenes in Atlantic cod (*Gadus morhua*) within the Gulf of Maine. BMC Genomics..

[19] Berg PR, Star B, Pampoulie C, Bradbury IR, Bentzen P, Hutchings JA (2017). Trans-oceanic genomic divergence of Atlantic cod ecotypes is associated with large inversions. Heredity..

[20] Sinclair-Waters M, Bradbury IR, Morris CJ, Lien S, Kent MP, Bentzen P (2018). Ancient chromosomal rearrangement associated with local adaptation of a post-glacially colonized population of Atlantic Cod in the northwest Atlantic. Mol Ecol.

[21] Berg PR, Star B, Pampoulie C, Sodeland M, Barth JM, Knutsen H (2016). Three chromosomal rearrangements promote genomic divergence between migratory and stationary ecotypes of Atlantic cod. Sci Rep..

[22] Sodeland M, Jorde PE, Lien S, Jentoft S, Berg PR, Grove H (2016). Islands of divergence in the Atlantic cod genome represent polymorphic chromosomal rearrangements. Genome Biol Evol..

[23] Pogson GH, Mesa KA, Boutilier RG (1995). Genetic population-structure and gene flow in the Atlantic cod *Gadus morhua*—a comparison of allozyme and nuclear RFLP loci. Genetics.

[24] Moen T, Hayes B, Nilsen F, Delghandi M, Fjalestad KT, Fevolden SE (2008). Identification and characterisation of novel SNP markers in Atlantic cod: evidence for directional selection. BMC Genet.

[25] Bradbury IR, Bowman S, Borza T, Snelgrove PV, Hutchings JA, Berg PR (2014). Long distance linkage disequilibrium and limited hybridization suggest cryptic speciation in Atlantic cod. PLoS One.

[26] Berg PR, Jentoft S, Star B, Ring KH, Knutsen H, Lien S (2015). Adaptation of low salinity promotes genomic divergence in Atlantic cod (*Gadus morhua* L.). Genome Biol Evol..

[27] Bangera R, Ødegård J, Nielsen HM, Gjøen HM, Mortensen A (2013). Genetic analysis of vibriosis and viral nervous necrosis resistance in Atlantic cod (*Gadus morhua* L.) using a cure model. J Anim Sci..

[28] Bangera R, Ødegård J, Præbel AK, Mortensen A, Nielsen HM (2011). Genetic correlations between growth rate and resistance to vibriosis and viral nervous necrosis in Atlantic cod (*Gadus morhua* L.). Aquaculture..

[29] Star B, Nederbragt AJ, Jentoft S, Grimholt U, Malmstrøm M, Gregers TF (2011). The genome sequence of Atlantic cod reveals a unique immune system. Nature.

[30] Langmead B, Salzberg SL (2012). Fast gapped-read alignment with Bowtie 2. Nat Methods.

[31] Li H, Handsaker B, Wysoker A, Fennell T, Ruan J, Homer N (2009). The sequence alignment/map format and SAMtools. Bioinformatics.

[32] Garrison E, Marth G. 2012. https://arxiv.org/abs/1207.3907. Haplotype-based variant detection from short-read sequencing. Accessed 23 Aug 2019.

[33] Malécot G (1948). Les mathématiques de l’hérédité.

[34] Weir BS, Cardon LR, Anderson AD, Nielsen DM, Hill WG (2005). Measures of human population structure show heterogeneity among genomic regions. Genome Res.

[35] Raymond M, Rousset F (1995). GENEPOP (version 1.2): population genetics software for exact tests and ecumenicism. J Hered..

[36] Weir BS, Cockerham CC (1984). Estimating F-statistics for the analysis of population structure. Evolution.

[37] Jombart T (2008). Adegenet: an R package for the multivariate analysis of genetic markers. Bioinformatics.

[38] Falconer DS, Mackay TFC (1996). Introduction to quantitative genetics.

[39] Rosenzweig BK, Pease JB, Besansky NJ, Hahn MW (2016). Powerful methods for detecting introgressed regions from population genomic data. Mol Ecol.

[40] Hohenlohe PA, Bassham S, Currey M, Cresko WA (2012). Extensive linkage disequilibrium and parallel adaptive divergence across three spine stickleback genomes. Philos Trans R Soc Lond B Biol Sci.

