# Supplementary material for: Strong selection pressures maintain divergence on genomic islands in Atlantic cod (Gadus morhua L.) populations
Source: Genet Sel Evol. 2019 Oct 29;51:61. doi: 10.1186/s12711-019-0503-5 (PMC6819574; doi:10.1186/s12711-019-0503-5)
Supplement: Supplementary file 1 — Additional file 1: Figure S1. Patterns of coancestry ($f$f) in all chromosomes (except 1, 2, 7 and 12) for the CC, NEAC, CC × NEAC and $f_{{{\text{CC}}\;{\&}\;NEAC}}$fCC&NEAC populations. The data provided represent values of coancestry smoothed by using a sliding window approach. Figure S2. Genetic differentiation coefficients ($F$FST) between populations CC and NEAC in all chromosomes (except 1, 2, 7 and 12). The showed values are genetic differentiation coefficients smoothed by using a sliding window approach. Figure S3. Private allele frequency in populations CC (left column) and NEAC (right column) in all chromosomes (except 1, 2, 7 and 12). A sliding window approach was used to smooth the private allele frequency. Figure S4. Principal components analysis in populations CC and NEAC in all chromosomes (except 1, 2, 7 and 12). First two principal components on the SNP data available. [file 12711_2019_503_MOESM1_ESM.doc]

**Figure S1** Patterns of coancestry (*f*) in all chromosomes (except 1, 2, 7 and 12) for the CC, NEAC, CC × NEAC and *f*_CC&NEAC_ populations.

**Figure S1 (continued)**

**Figure S1 (continued)**

**Figure S1 (continued)**

**Figure S1 (continued)**

**Figure S1 (continued)**

**Figure S1 (continued)**

**Figure S1 (continued)**

**Figure S1 (continued)**

**Figure S1 (continued)**

**Figure S2** Genetic differentiation coefficients (*F_ST_*) between populations CC and NEAC in all chromosomes (except 1, 2, 7 and 12).

**Figure S2 (continued)**

**Figure S2 (continued)**

**Figure S3** Private allele frequency in populations CC (left column) and NEAC (right column) in all chromosomes (except 1, 2, 7 and 12).

**Figure S3 (continued)**

**Figure S3 (continued)**

**Figure S3 (continued)**

**Figure S3 (continued)**

**Figure S4** Principal components analysis in populations CC and NEAC in all chromosomes (except 1, 2, 7 and 12).

**Figure S4 (continued)**

**Figure S4 (continued)**
